# Sodium Valproate Incompatibility with Parenteral Nutrition Admixtures—A Risk to Patient Safety: An In Vitro Evaluation Study

**DOI:** 10.3390/pharmaceutics14020371

**Published:** 2022-02-07

**Authors:** Ludwika Piwowarczyk, Szymon Tomczak, Patryk Antkowiak, Anna Jelińska, Maciej Stawny

**Affiliations:** Department of Pharmaceutical Chemistry, Poznan University of Medical Sciences, 6 Grunwaldzka, 60-780 Poznań, Poland; lpiwowarczyk@ump.edu.pl (L.P.); szymon.tomczak@ump.edu.pl (S.T.); anakreontyk1994@gmail.com (P.A.); ajelinsk@ump.edu.pl (A.J.)

**Keywords:** sodium valproate, compatibility, safe therapy, interaction, medical errors

## Abstract

Epilepsy is defined as a group of concerning problems related to the nervous system; its defining feature is a predisposition to epileptic seizures. The frequency of seizures in intensive care units (ICU) ranges from 3.3% to 34%, and ICU antiepileptic treatment is routine practice. The administration of drugs through the same infusion line is not recommended but is common clinical practice, especially in ICU. Incompatibilities between parenteral drugs and between drugs and parenteral nutrition admixtures (PNAs) are common medical errors and pose risks to patient safety. The co-administration of drugs must always be confirmed and clearly defined. The simultaneous infusion of sodium valproate (VPA, drug used to treat seizures and epilepsy) with parenteral PNAs has not yet been studied. During the experiment reported in this study, a visual control, pH, osmolality, zeta potential, particle size, polydispersity index, and turbidity were measured. The conducted research shows that the lipid emulsion composition has a significant influence on drug–PN (drug–parenteral nutrition) compatibility. The acceptance criteria were met only for PNs containing omega-3-acid-triglycerides (Omegaflex special and peri). The second fraction of particles above 1000 nm was observed for most of the tested PNAs (Lipoflex special, Lipoflex peri, Kabiven, SmofKabiven, Kabiven Peripheral, and Olimel Peri N4E), which disqualifies their simultaneous administration with VPA.

## 1. Introduction

Epilepsy may be described as a neurological disease. Due to the complexity of its characteristics, it is often defined as a group of problems related to the nervous system, whose defining feature is a predisposition to epileptic seizures [1]. It is estimated that up to 50 million people worldwide are affected by epilepsy [2]. Based on Healthcare Cost and Utilization Project (HCUP) data, epilepsy or convulsion diagnoses led to 280,000 hospital admissions and more than 1 million emergency department visits, with aggregate hospital costs of approximately $2.5 billion [3]. The frequency of seizures in intensive care units (ICU) ranges from 3.3% to 34% [4]. In ICUs, antiepileptic treatment is routine due to a wide range of risk factors, including common diagnoses such as brain tumor, head injury, stroke, history of epileptic seizures, electrolyte disturbances, hypoglycemia, infections, and drug overdose or discontinuation [5]. Changes in patient physiology and the physical properties of drugs can affect the rate and extent of enteral drug absorption by critically ill patients. In addition, gastrointestinal malabsorption conditions due to reduced blood flow, gut atrophy, motor dysfunction, and interaction with enteral nutrition necessitate a parenteral route of administration [6].

Patients admitted to ICU suffer from life-threatening conditions with several morbidities and dysfunctions. It has been found that malnutrition has a negative impact on the incidence of postoperative complications, length of hospitalization, and mortality [7,8,9,10,11,12,13]. Hence, it is necessary to introduce a nutritional intervention individualized to the needs and condition of the patient. Parenteral nutrition (PN) is only used in patients for whom enteral administration is impossible or does not meet all nutritional needs [7,14].

Parenteral nutrition (PN) therapy is an unquestionable achievement of twentieth-century medicine that allows patients with severe impairment or gastrointestinal tract obstruction to survive. The infusion of parenteral nutrition admixtures (PNAs) lasts over 20 h. Other parenteral medications should be administered separately from PNAs. Thus, polypharmacy in patients receiving parenteral nutrition may cause difficulties in planning medication schedules, especially when the number of drugs exceeds the number of available access lines. This problem concerns critical care and cancer patients, who often need the simultaneous administration of multiple intravenous drugs. Although PN plays a significant role in many cases, there is a risk of interaction when PNA is co-administered with drugs. This kind of combined therapy may lead to drug–drug or drug–nutrient interactions, such as discoloration, precipitation, destabilization of the drug, or lipid emulsion [15,16,17,18,19,20]. Chemical and physical interactions between a drug and PNA can occur prior to administration to a patient. Nevertheless, it should be noted that PNA may affect the pharmacokinetic (PK) and pharmacodynamic (PD) parameters of drugs such as plasma protein binding and enterohepatic or renal transportation [21,22]. Plasma protein binding may be influenced by increased free fatty acid in serum. This type of interaction was observed for valproic acid and phenytoin, manifesting as an increase in the free fraction of the drug. Free fatty acids displaced drugs from their albumin-binding sites [23,24,25,26]. Bailey et al. [27] performed in vitro evaluation of some drugs, including anticonvulsants and five PNA compositions. Phenytoin, phenobarbital, and valproic acid were observed to be less bound to human serum in the presence of PN. The opposite effect was observed with carbamazepine. These data suggested that PNA administration may significantly alter the free fraction of drugs. At the same time, it should be highlighted that in vitro studies of PK–PD interactions have some limitations and may not reflect the conditions of drug–PNA interactions that occur in vivo.

Our study focused on physical and chemical interactions between PNA and VPA solutions. The compatibility between sodium valproate (VPA) and intravenous drugs was investigated previously [28,29], but there are still no data regarding the compatibility of VPA with lipid emulsion. Therefore, the purpose of our research was to improve the safety of the administration of VPA solutions with eight different ready-to-use (RTU) parenteral nutrition admixtures and determine the possible interactions between the studied drugs.

## 2. Materials and Methods

### 2.1. Materials

In total, 400 mg/4 mL (Sanofi-Aventis, Paris, France; LOT A90641X, EXP 05.2022) sodium valproate, an antiepileptic drug, with the brand name Depakine^®^, was purchased from a local market and used in this study. One ampule contains 400 mg of sodium valproate, which must be dissolved in normal saline or glucose before administration. In this study, we used normal saline solution (B. Braun Melsungen AG, Melsungen, Germany; LOT 19381450, EXP 08.2022) and 5% glucose (B. Braun Melsungen AG, Melsungen, Germany; LOT 19257406, EXP 05.2022). 

As the RTU, three chambers of PNAs were analyzed in this study, i.e., Omegaflex special, Lipoflex special, Lipoflex peri, Omegaflex peri (B. Braun Melsungen AG, Melsungen, Germany), Kabiven, SmofKabiven, Kabiven Peripheral (Fresenius Kabi AB Uppsala, Uppsala, Sweden), and Olimel Peri N4E (Baxter, Warsaw, Poland). The use of this broad spectrum of PNA compositions was intended to determine the influence of the ingredients on possible interactions with drug solutions. These PNAs differed in their composition, their source of lipid (fish, soy bean, olive oil), energy, amino acid, and nitrogen content, their and route of administration (peripheral and central access) (Table 1). In clinical practice, vitamins and trace elements (TE) are supplemented before the administration of PNA to the patient’s vein. Due to the possible effect on the stability of the drug, the test of the PNA was performed with vitamins and TE, in accordance with the manufacturer’s requirements. As vitamin sources, we used: Viantan B. Braun Melsungen AG, Melsungen, Germany) added to Omegaflex special, Lipoflex special, Lipoflex peri, Omegaflex peri; Vitalipid N Adult and SOLUVIT N (Fresenius Kabi AB Uppsala, Sweden) added to Kabiven, SmofKabiven, Kabiven Peripheral; and Cernevit (Baxter, Warsaw, Poland) added to Olimel PeriN4E. Tracutil (B. Braun Melsungen AG, Melsungen, Germany) was the source of TE.

### 2.2. Methods

This study assessed the compatibility of Depakine 400 mg/4 mL with eight PNAs. The study simulated the contact of sodium valproate with each PNA, as would occur in the case of the simultaneous supply of both ingredients via a Y-type connector. Compatibility tests were performed in a static manner. The drug and the PNA were mixed in the appropriate proportion in test tubes and then analyzed. The choice of the volume proportion depends primarily on the hypothetical volume ratio in which the two fluids mix in the joint line of the Y-site catheter. For this purpose, the ratios were calculated based on the flow rates and duration of administration. The extreme minimum and extreme maximum were used and 5:5 was added for comparison purposes. Table 2 presents the calculated ratios. The chosen ratios for the study are bolded.

The determination of the drug’s compatibility with the PNA was performed by a series of parallel measurements, i.e., pH, osmolality, polydispersity index (PDI), visual control, zeta potential, and particle size. Additionally, the turbidity of the prepared mixtures was measured to determine the precipitation of sediment. All measurements were performed immediately after mixing the drug and the PNA (t_1_ = 0 h) and after four hours (t_2_ = 4 h) storage and exposure to light at room temperature. The second time point, significantly exceeding the real-time exposure to the PNA drug, was determined by capturing the interactions over time. For all measurements of the samples containing the lipid emulsion (excluding turbidity measurements), the PNA with vitamins and TE was used as a reference test.

#### 2.2.1. Visual Control

Visual control aims to identify with the naked eye the changes taking place in the mixture, i.e., color changes, sedimentation or precipitation of insoluble substance, delamination of the lipid emulsion. In accordance with the European Pharmacopeia [30], this analysis was performed by two investigators on a white and a black background. It was performed for both series: drug-free PNA (blanks) and combinations of drug and PNA. An admixture that is homogeneous, uniformly colored, and free of solids meets the requirements.

#### 2.2.2. pH Measurement

The measurement of pH is a simple, quick method that makes it possible to capture changes in the composition of a PNA. The measurement was carried out by measuring the pH value of the 10 mL mixture consisting of an appropriate volume ratio of PNA and VPA solution using a Mettler Toledo Seven Compact pH/ionS220 pH meter. Significant changes in the pH of the PNA may indicate its decomposition, e.g., a lowering of the pH by increasing the concentration of free fatty acids as a product of triglyceride hydrolysis. The pH value additionally affects the compatibility of drugs added together with PNAs. Excessively high or low changes in pH could cause phase separation of lipid emulsion or the precipitation of drugs.

The pH of the PNA should not change during the test (4 h) by 0.2, and the change after mixing with the drug should not be greater than the ΔpH = 0.5 [18,31].

#### 2.2.3. Osmolality Measurement

The measurement of osmolality aims to determine the content of osmotically active substances. The concentration of ions decreases in the solution during the precipitation, manifesting as a significant difference between the measurements. The osmolality values were recorded using an osmometer, based on the principle of the freezing-point depression method (TridentMed, Warsaw, Poland). A total of 100.0 µL of sample was transferred into an OSMO-KRIO cuvette, and then analyzed. The acceptance limit for osmolality changes was set as <5% [16,31].

#### 2.2.4. Polidispersity Index, MDD and Zeta Potential Measurements

These three measurements were determined using a Zetasizer Nano ZS (Malvern Instruments, Malvern, UK). The measurement of zeta wis performed through charge migration in the external electric field. By contrast, laser analysis is performed to measure the size of the lipid emulsion particle (expressed as mean emulsion droplet diameter—MDD and PDI). In total, 1 mL of PNA sample was diluted with 9 mL of water, and transferred into a U-shaped cuvette with a golden electrode. This special equipment made it possible to perform parallel determinations.

#### 2.2.5. Turbidity Measurement

A direct test of lipid emulsion is not possible due to the white color of PNA. Because sediment would potentially be formed as a result of the reaction of ions with drug molecules, the volume fraction of the lipid emulsion was replaced by the addition of water for injections (B. Braun Melsungen AG, Melsungen, Germany). This admixture was supplemented only by TE, and the vitamin preparations were omitted due to their color and their presence in the form of a lipid emulsion.

Turbidity was measured using a TU52000 Laboratory Laser Turbidimeter (Hach Company, Loveland, CO, USA). A three-stage calibration always preceded the tests using standard solutions with the values of 10, 20, and 600 NTU—nephelometric turbidity unit (Hach Company, Loveland, CO, USA). A total of 10 mL of sample in glass cuvettes was placed in a turbidimeter cell; next, measurements were performed in triplicate. The results are expressed as the mean value with standard deviation. A change in turbidity after adding the drug and during the evaluation period should not exceed a value of 0.5 NTU. Greater changes would indicate the incompatibility of the tested admixture with the drug solution [32,33,34].

#### 2.2.6. Statistical Analysis

Repeated-measures analyses of variance (ANOVAs) were carried out for statistical analysis using Statistica 12 software (Statistica, Tulusa, OK, USA). The a priori level of significance was *p* < 0.05.

## 3. Results

### 3.1. Parenteral Nutrition Admixture without Drug Solutions—Reference Samples

As a result of the compatibility tests carried out, no changes indicating the decomposition of the emulsion were observed with the naked eye. The drug-free admixtures remained stable throughout the experiment and no disturbance changes were observed, which could indicate the decomposition of the lipid emulsion. The results for the PNA without drug solution are presented in Table 3. The pH value ranged from 5.51 to 6.45 for Lipoflex special and Olimel Peri N4E, respectively. The turbidity of the non-lipid part was low, with a maximum value of 0.328 ± 0.025 (Omegaflex Special). High differences in osmolality refer to the route of administration. The PNAs with osmolality >1000 mOsm/kg are dedicated to central vein administration, whereas PNAs with lower osmolality (<1000 mOsm/kg) are used for peripheral administration. The zeta potential values were negative and ranged from −17.20 to −8.35 mV (Olimel Peri N4E and Lipoflex special, respectively). All the PNAs were homogenous, the PDI was close to 0.1, and the crucial parameter, i.e., MDD, was below the USP limit of 500 nm; to be more specific, it ranged from 240.37 to 277.67 nm. We did not detect any significant changes during the experiment, which proved the required stability of the PNAs.

### 3.2. Parenteral Nutrition Admixture with Drug Solutions

Mixing the drug solution and the PNA can cause interactions, including those that are invisible to the naked eye. The visual analysis did not provide data on the negative effects of such a mixture. Neither phase separation nor color changes were observed. Mixing PNA with drug solutions resulted in minimal changes in pH, not exceeding 0.2 units. The highest difference (ΔpH = 0.17) was observed for Lipoflex Special (increase) and Omegaflex Peri (decrease) in the ratio 7:3 with glucose. Another analyzed parameter was osmolality, which mainly depends on the volumes of PNA-containing electrolytes. Drug– PNA admixtures achieve decreasing osmolality values with decreasing PNA content. The largest difference was observed for Lipoflex Special. The decrease was as much as 1275 mOsm/kg after mixing in a ratio of 7:3 with normal saline, while changes during the four hours of the experiment were slight and constituted no more than 2%.

**Figure 1:**
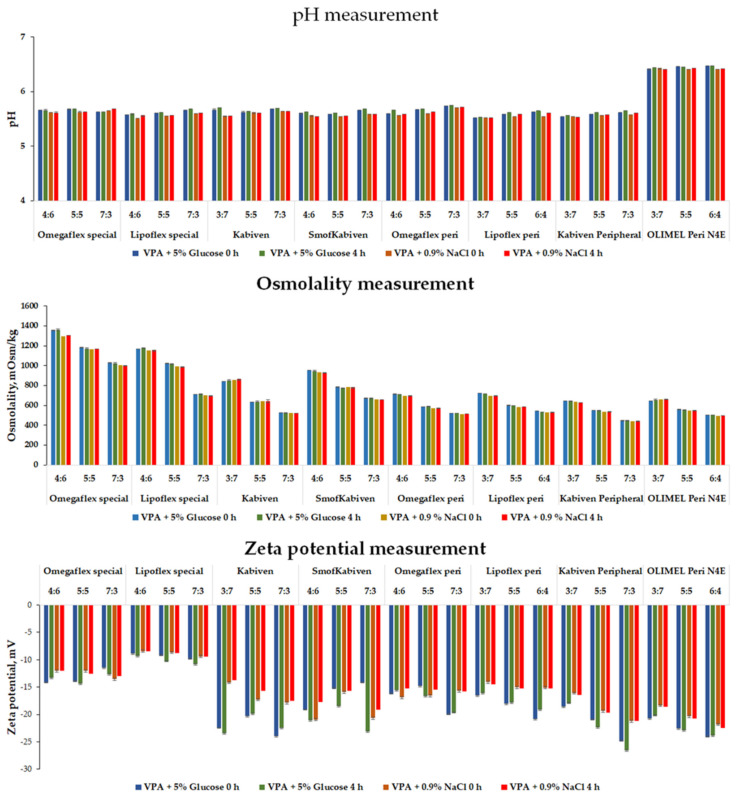
Results for pH, osmolality, and zeta potential measurements for mixed PNA with drug solutions (VPA + 5% Glucose or 0.9% NaCl).

The zeta potential values decreased when the drug’s solution was mixed with PNA. The greatest changes were observed for the sample containing Kabiven mixed in a 7:3 ratio with VPA glucose solution, and it dropped by as much as −12.83 mV to −23.43 mV. The zeta potential of the tested combinations’ drug mixture ranged from −26.6 mV for Kabiven Peripheral mixed with VPA glucose solution in a of ratio 7:3 to −8.41 for Lipoflex Special mixed with normal saline VPA in a ratio of 4:6. Despite the wide variation between the tested admixtures, all the values were negative. These changes positively influenced the stability of the lipid emulsion due to the increased interaction of the emulsion particles with each other. Figure 1 summarizes the results from the pH, osmolality, and zeta potential measurements.

**Figure 2:**
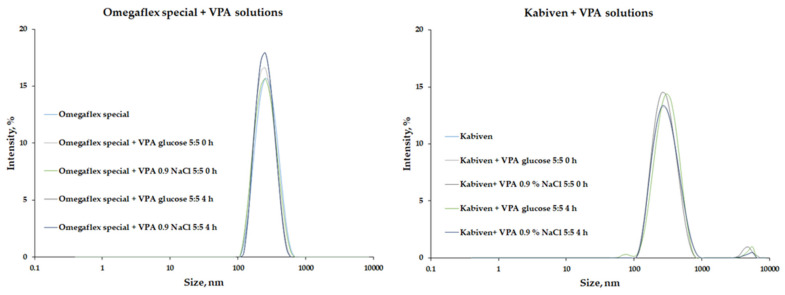
Results of size distribution for Omegaflex special (samples without second fraction) and Kabiven (samples with second fraction of lipid droplets higher than 1000 nm). VPA—sodium valproate.

A key parameter for safe therapy is the lipid emulsion’s particle size (MDD). The addition of drug solutions to the PNA caused slight particle size changes, an average increase of 13.3 for Lipoflex mixed with 0.9% NaCl solution of VPA. On the other hand, the presence of the second fraction, i.e., lipid particles larger than 1000 nm, was observed for most of the tested admixtures. Only combinations with Omegaflex and Omegaflex peri, i.e., mixtures based on lipids rich in omega-3-acid triglycerides, were free from the second fraction (Figure 2). The PDI values ranged from 0.088 to 0.203, and for PDI values greater than 0.13, the second fraction was observed. This increase in PDI value resulted from a change in the homogeneity of the lipid–water system. Table 4 summarizes the results for MDD and the presence of the second fraction of emulsion particles.

The turbidity measurements were performed on a lipid-free admixture supplemented only by TE. After the addition of the drug solution, the changes were below the acceptance limit (0.5 NTU), with a maximum increase of 0.324 NTU for Omegaflex Special mixed with 0.9% NaCl solution in a ratio of 7:3. During the evaluation period, the changes were lower, with a change in turbidity of up to 0.122 NTU.

## 4. Discussion

Parenteral nutrition admixtures consisted of numerous different elements (water, amino acids, electrolytes, trace elements, vitamins, and lipids). This rich composition makes them prone to interaction when mixed with a parenteral solution, including drug solutions. Based on the effect and place of action, interactions may be divided into physicochemical interactions, occurring during drug preparation and administration, and pharmacological interactions, which take place in vivo and alter drug action. Reactions between soluble components and interactions with the container are forms of physicochemical incompatibility [35]. Other known interactions between PNA and anticonvulsants are affected by pharmacokinetics, such as the displacement of drugs from their albumin-binding sites, which may have significant consequences. Only free drugs can produce the pharmacological action by crossing the plasma membrane and binding with the receptors. The increase in free drug concentration may cause drugs to reach toxicity levels and the occurrence of side effects. The plasma protein binding of valproic acid ranges from 90 to 95% [36]. The use of free valproic acid concentration instead of total serum concentration in the management of epilepsy and the avoidance of unwanted side effects [37]. This type of interaction was observed for valproic acid and phenytoin. Zimmerman et al. [25] reported an increase in the free fraction of valproic acid in serum as a result of the increased concentration of free fatty acids. Likewise, Dutkiewicz et al. [26] observed a similar change in phenytoin concentration for hypercholesterolemia and in a mixed hyperlipidemia population. Similarly, the effect may have a high glucose concentration. Doucet et al. observed a significant decrease in phenytoin protein binding in serum from diabetics. The effect on drug binding to site II of human serum albumin level may be modified by L-tryptophan, which is a component of PNAs [21]. All the studied PNAs contained this amino acid, ranging from 0.42 g/1000 mL to 1.02 g/1000 mL for OlimelPeri N4E and Smofkabiven, respectively. The components of artificial nutrition may have an impact on drug adsorption intensities and on drug concentration. Van Den Bergh et al. [38] reported a case study of a clinically significant decrease in VPA serum concentration after initializing the administration of an enteral protein supplement. Polytherapy with some anticonvulsant drugs (phenytoin, carbamazepine, and oxcarbazepine) that are inductors of hepatic enzymes may lead to a decrease in the amount of drug metabolized in the liver by cytochrome P450 enzymes [23]. Despite the known interactions in the PK phase with VPA [21,24,25], there is still no evidence for a significant effect on patient conditions due to the lack of prospective, randomized, and controlled trials [21].

Given the above, this work focuses on capturing physicochemical interactions. Sodium valproate belongs to the first-line drugs used to treat seizures and epilepsy. According to a summary of its characteristics, Depakine is administered through continuous infusion in solutions of 0.9% NaCl and 5% glucose, with an infusion rate ranging from 1 to 2 mg/kg body weight/h. The PNAs selected for testing in this study were eight ready-to-use PNAs widely used in clinical practice worldwide. The PNAs differed in caloric intake, nitrogen and lipid content, and lipid source. Additionally, this study was designed to capture electrolyte influence on stability using PNA for central (Lipoflex Special, Omegaflex special, Kabiven and Smofkabiven) and peripheral access (Lipoflex peri, Omegaflex peri, Kabiven peripheral, and Olimel peri N4E).

In the case of unknown compatibility, parenteral drugs must be administered through separate catheter lines. The interaction risk concerns both the reduction of the drug’s effect and the influence on the stability of the lipid emulsion. Incompatibilities between parenteral drugs are common medical errors and pose a high risk to patient safety. Considering the above, the co-administration of drugs must always be confirmed and clearly defined. The simultaneous infusion of sodium valproate with PN admixtures has not yet been studied, to the best of our knowledge. However, the possibility of the combined administration of VPA with some drugs has been determined [28,29]. Frank et al. [29] studied the physical compatibility of valproate sodium injection with dobutamine and dopamine and proved the physical stability of this combination. Rashed et al. [28] tested the compatibility of VPA in concentrations of 2 mg/mL and 20 mg/mL with thirteen medications commonly used in the acute care setting. VPA was incompatible only with diazepam, midazolam and phenytoin sodium, which was manifested in precipitation.

Until now, no official compendium or harmonized methodology has been established to evaluate PNA stability. Based on the research data and our own experience, we used different analytical methods which allowed us to detect changes in the physicochemical properties of VPA-RTU samples [15,16,17,18,31,32,39,40,41,42,43,44,45,46]. Despite the duration of the VPA infusion of approximately a few hours and of the PNA from 16 to 24 h, the real-time contact in a common line of Y-site connector lasted only a few minutes. Compatibility studies were performed at two endpoints (0 h and 4 h). The choice of the second endpoint (4 h) was intended to capture time-dependent changes in the studied samples. In the course of the experiment, a visual control along with the pH, osmolality, zeta potential, particle size, PDI, and turbidity of the lipid-free sample measurements were performed. The visual control of the PNA samples concluded that the admixture was free of sediment and signs of emulsion breakdown, both for mixtures with and without the addition of sodium valproate solutions. The pH measurements in the stability study of lipid emulsion are utilized to capture the production of free lipid acid formed in the rancidification process (acid-base changes) or precipitation. An excessively difference in pH after mixing may cause a precipitation of drug or calcium phosphate. A decrease in pH below 5.0 may be susceptible to phase separation of the emulsion [20]. However, it is not the only factor contributing to the emulsion’s breakdown. Buffering components such as amino acids and electrolytes prevent the properties of the emulsion from lowering the pH. The mixing of PNAs with VPA solutions had a minimal effect on the pH of the resulting liquids. Minimal changes in pH, not exceeding 0.2 units, did not exceed the acceptance criterion. In addition, the turbidity test of the lipid-free samples also showed no sign of sediment or precipitation. None of the samples tested exceeded the difference during the evaluation period, with 0.5 NTU as the acceptance criterion. The osmolality of the PNA without drugs was consistent with the route of administration, high osmotic PNAs for central access, and low (below 1000 mOsm/kg) for peripheral access. Nevertheless, no changes in osmolality were observed during the experiment, and the major change was only 1.9% for Olimel Peri N4Eolmel mixed with VPA glucose solution in a ratio of 3:7. The peripheral administration of fluids with high osmolality (>1000 mOsm/kg) may endanger patients’ lives; it is related to a high risk of phlebitis and may lead to dehydration and the shrinking of blood cells [47].

The zeta potential is a parameter related to the stability of the lipid emulsion. In the case of PNA, it takes values below zero due to emulsifiers, most often phospholipids. The anionic fractions of phospholipids and intercalated oleate ions are mostly responsible for the negative charge. The zeta potential defines the nature of electrostatic interactions between the emulsion particles and the medium. It depends on the pH and the concentration of electrolytes. Electrolytes modify the zeta potential by adsorbing to the droplet surface and screening the droplet charges by, for example, adding the divalent ions, i.e., calcium; magnesium reduces it [48]. The degrading lipid emulsion takes increasing zeta potential values up to the point of zero charge [48]. All of the tested admixtures, both the reference sample and samples with the addition of drug solutions, had negative values, varying from −17.20 mV to −8.35 mV for the reference sample and from −26.6 mV to −8.41 mV for the PNAs mixed with drug solutions. During the evaluation period, no significant changes in zeta potential were observed.

The particle size and distribution of lipid emulsion play a key role in safe therapy. Due to their crucial role, they have been included among the critical quality attributes (CQAs) for liposome drug products by the Food and Drug Administration [49]. The United States Pharmacopeia, in Chapter <729> [50], recommends two methods for determining particle size: Method I and Method II. Method I involves dynamic light scattering (DLS) techniques used to determine MDD in lipid emulsions. Method II is based on a light obscuration (LO) or light extinction (LE) method that is used to determine the extent of the large-diameter droplet tail (PFAT). In addition, lipid droplets with diameters larger than 500 nm in PNA should also be taken into account because they are associated with the emulsion stability and the safety of parenteral therapy. Another parameter connected with lipid droplet size, and recommended by IUPAC, is PDI (dispersity) [51], which provides information on the broadness of the droplet size distribution. A small PDI of <0.2 indicates a narrow and concentrated particle size distribution and, thus, better stability against destabilization [52,53], whereas a PDI above 0.7 indicates a very broad size distribution [54]. A PDI <0.3 indicates a homogenous population of phospholipid vesicles and is considered acceptable in liposome and nanoliposome formulations [55,56]. PDIs for reference samples (PNAs without drug addition) were close to 0.1 and ranged from 0.096 to 0.120. Additionally, the MDDs of the mentioned samples were below the USP limit (240.37–277.07 nm), and only one fraction of lipid droplets was detected. By contrast, the second fraction was detected after adding VPA solution in the majority of samples. The PDI values were higher than 0.13 for all the samples with a detected second lipid particle fraction. Only the Omegaflex special and Omegaflex peri samples were without the second fraction. These two PNAs are distinguished by a different composition of lipid emulsion, which is rich in omega-3-acid triglycerides. According to some available data, PNA with PDI values higher than 0.13 meet the criteria for parenteral administration [46,57,58].

## 5. Conclusions

The administration of drugs through the same infusion line is not recommended, but is common clinical practice, especially in ICU. This study shows that the composition of the lipid emulsion has a significant influence on drug–PN compatibility. The second fraction of particles above 1000 nm was observed for most of the tested PNAs, which disqualifies their simultaneous administration with VPA. The acceptance criteria were met only for PNs containing, among others, omega-3-acid triglycerides. Based on the results that were used to characterize the different physicochemical properties of the VPA-PN samples, it is possible to recommend the simultaneous infusion of valproate sodium with Omegaflex special and Omegaflex peri, maintaining used drug concentrations, infusion rate and fluids. At the same time, it should be emphasized that combining valproate sodium with Lipoflex special, Lipoflex peri, Kabiven, SmofKabiven, Kabiven Peripheral, and Olimel Peri N4E in one infusion line may cause medical consequences related to the introduction of lipid particles larger than 1000 nm into the bloodstream.

## Figures and Tables

**Table 1:** Composition comparison of studied parenteral nutrition admixtures (volume 1000 mL).

PNA		Total Energy (kcal)	Glucose (g)	Lipid (g)	MCT ^1^ (g)	Soya-Bean Oil (g)	Fish Oil (g)	Olive Oil (g)	Acids Ω-3 (g)	Amino Acids (g)	Nitrogen (g)	Vitamin Source
Central access	Omegaflex special	1184.0	144.0	40.0	20.0	16.0	-	-	4.0	56.0	8.0	Viantan
	Lipoflex special	1184.0	144.0	40.0	20.0	20.0	-	-	-	56.0	8.0	Viantan
	Kabiven	909.1	97.4	39.0	-	39.0	-	-	-	33.1	5.3	V + S ^2^
	SmofKabiven	1083.3	126.6	37.9	11.4	11.4	5.7	9.6	-	50.8	8.1	V + S ^2^
Peripheral access	Omegaflex peri	764.0	64.0	40.0	20.0	16.0	-	-	4.0	32.0	4.6	Viantan
	Lipoflex peri	764.0	64.0	40.0	20.0	20.0	-	-	-	32.0	4.6	Viantan
	Kabiven Peripheral	694.4	67.4	35.4	-	35.4	-	-	-	23.6	3.8	V + S ^2^
	Olimel Peri N4E	700.0	75.0	30.0	-	24.0	-	6.0	-	25.3	4.0	Cernevit

^1^ MCT—medium-chain triglycerides. ^2^ V + S—Vitalipid N adult and Soluvit N.

**Table 2:** The calculated volume ratio between drug solutions and PNA.

Parenteral Fluids	Infusion Rate, mL/h		Calculated Ratio VPA:PNA			
	Minimum	Maximum				
Omegaflex special	79	119	5:5	7:3	4:6	6:4
Lipoflex special	79	119	5:5	7:3	4:6	6:4
Kabiven	75	182	5:5	7:3	3:7	5:5
SmofKabiven	82	140	5:5	7:3	4:6	6:4
Omegaflex peri	78	175	5:5	6:4	3:7	5:5
Kabiven Peripheral	80	259	5:5	7:3	3:7	4:6
Lipoflex peri	78	175	5:5	7:3	3:7	5:5
Olimel Peri N4E	104	224	5:5	6:4	3:7	4:6
Sodium valproate solutions	88.2	176.4	- - -			

**Table 3:** Results for RTU-PNAs after activation at 0 h.

PNA	pH ± SD	Turbidity ± SD (NTU)	Osmolality ± SD (mOsm/kgH_2_O)	Zeta Potential ± SD (mV)	MDD ± SD (nm)	PDI ± SD
Omegaflex special	5.58 ± 0.01	0.328 ± 0.025	1925.00 ± 0.00	−11.70 ± 0.53	249.27 ± 3.25	0.096 ± 0.009
Lipoflex special	5.51 ± 0.01	0.133 ± 0.004	1972.00 ± 0.00	−8.35 ± 0.55	267.27 ± 3.76	0.102 ± 0.024
Kabiven	5.57 ± 0.01	0.169 ± 0.009	1044.00 ± 0.00	−10.60 ± 0.46	277.67 ± 1.96	0.116 ± 0.008
SmofKabiven	5.53 ± 0.01	0.169 ± 0.009	1596.00 ± 0.00	−12.00 ± 0.44	240.37 ± 2.73	0.106 ± 0.011
Omegaflex peri	5.48 ± 0.01	0.118 ± 0.001	903.00 ± 0.00	−15.07 ± 0.61	244.80 ± 2.91	0.112 ± 0.008
Lipoflex peri	5.74 ± 0.01	0.091 ± 0.001	938.15 ± 6.36	−14.47 ± 0.59	257.40 ± 0.70	0.092 ± 0.017
Kabiven Peripheral	5.63 ± 0.01	0.180 ± 0.025	808.00 ± 0.00	−15.03 ± 0.51	267.50 ± 0.95	0.120 ± 0.018
Olimel Peri N4E	6.45 ± 0.01	0.140 ± 0.002	846.00 ± 0.00	−17.20 ± 0.17	257.33 ± 1.45	0.111 ± 0.019

**Table 4:** The size of lipid droplet (MDD ± SD) in comparison to occurrence of the second fraction of lipid droplet.

PNA		VPA Solution in 5% Glucose				VPA Solution in 0.9% NaCl			
		t_1_ = 0 h		t_2_ = 4 h		t_1_ = 0 h		t_2_ = 4 h	
Omegaflex special	4:6	241.2 ± 4.6		243.5 ± 3.2		239.9 ± 2.6		243.0 ± 2.6	
	5:5	239.7 ± 3.6		241.3 ± 1.4		242.2 ± 1.6		241.9 ± 1.0	
	7:3	241.8 ± 8.9		247.4 ± 1.3		238.3 ± 4.8		240.3 ± 0.4	
Lipoflex special	4:6	264.0 ± 7.8	+	257.8 ± 1.0		259.5 ± 1.4		258.1 ± 1.8	
	5:5	262.1 ± 4.8	+	261.0 ± 1.0		262.6 ± 2.7		259.0 ± 2.7	
	7:3	255.3 ± 6.9	+	258.4 ± 3.1		256.7 ± 3.8		254.0 ± 2.9	+
Kabiven	3:7	277.1 ± 2.8	+	279.8 ± 3.8	+	277.8 ± 4.3		276.4 ± 3.9	
	5:5	275.0 ± 1.8	+	276.6 ± 5.1	+	274.6 ± 5.3	+	273.9 ± 2.7	+
	7:3	274.5 ± 2.5	+	277.6 ± 4.0	+	276.6 ± 6.1	+	277.0 ± 3.9	+
SmofKabiven	4:6	231.5 ± 2.4		236.2 ± 6.0		236.3 ± 3.6		243.8 ± 2.9	+
	5:5	235.5 ± 3.2		237.6 ± 6.2		234.9 ± 4.5	+	234.3 ± 2.9	+
	7:3	242.8 ± 8.1		234.6 ± 1.6		232.2 ± 1.8		234.8 ± 5.2	
Omegaflex peri	4:6	246.2 ± 3.8		249.2 ± 2.4		248.2 ± 4.4		248.2 ± 0.8	
	5:5	249.4 ± 1.9		241.5 ± 2.4		249.4 ± 4.9		246.5 ± 3.0	
	7:3	242.5 ± 1.8		247.8 ± 0.7		242.5 ± 3.9		243.7 ± 5.1	
Lipoflex peri	3:7	261.4 ± 2.5		257.1 ± 3.6		259.3 ± 3.4		254.4 ± 1.6	
	5:5	257.6 ± 2.6		257.0 ± 2.4	+	256.3 ± 2.1		252.8 ± 2.8	
	6:4	258.5 ± 1.3		256.0 ± 1.2	+	256.5 ± 1.2		255.2 ± 2.1	
Kabiven Peripheral	3:7	269.0 ± 5.0	+	269.3 ± 1.9		268.1 ± 2.9		267.5 ± 0.7	
	5:5	266.9 ± 1.6	+	272.0 ± 3.4		270.6 ± 5.2	+	267.9 ± 1.6	+
	7:3	272.0 ± 2.1	+	274.1 ± 2.2	+	263.4 ± 2.0	+	274.5 ± 2.0	+
Olimel Peri N4E	3:7	263.0 ± 5.0		257.4 ± 1.7		258.3 ± 5.1		257.1 ± 4.7	
	5:5	256.1 ± 3.3	+	254.9 ± 3.9		261.1 ± 6.1	+	258.4 ± 5.4	+
	6:4	266.9 ± 4.9	+	258.9 ± 3.1	+	257.2 ± 3.4		255.3 ± 4.7	

+—detected lipid droplets higher than 1000 nm (second fraction).

## Data Availability

The data presented in this study are available upon request from the authors.
